# Bis[5-(4-bromo­phen­yl)-4-(*tert*-but­oxy­carbon­yl)pyrrolidine-2-carboxyl­ato]copper(II) dihydrate

**DOI:** 10.1107/S1600536811043893

**Published:** 2011-10-29

**Authors:** Konstantin V. Kudryavtsev, Andrei V. Churakov, Ozdemir Dogan

**Affiliations:** a Department of Chemistry, M.V. Lomonosov Moscow State University, Leninskie Gory 1/3, Moscow 119991, Russian Federation; b Institute of General and Inorganic Chemistry, Russian Academy of Sciences, Leninskii prosp. 31, Moscow 119991, Russian Federation; c Department of Chemistry, Middle East Technical University, Ankara 06531, Turkey

## Abstract

In the title compound, [Cu(C_16_H_19_BrNO_4_)_2_]·2H_2_O, the Cu^II^ ion resides on an inversion centre and is coordinated by two O and two N atoms from two enanti­omeric 5-(4-bromo­phen­yl)-4-(*tert*-but­oxy­carbon­yl)pyrrolidine-2-carboxyl­ate ligands in a distorted square-planar geometry. The relative stereochemistry of the three stereogenic C atoms in each ligand has been determined. In the crystal, inter­molecular N—H⋯O and O—H⋯O hydrogen bonds link the mol­ecules into layers parallel to the *bc* plane. The crystal studied was twinned by pseudo­merohedry with twin fractions of 0.719 (3) and 0.281 (3).

## Related literature

For details of the ligand synthesis, see: Kudryavtsev *et al.* (2006 ▶, 2010 ▶).
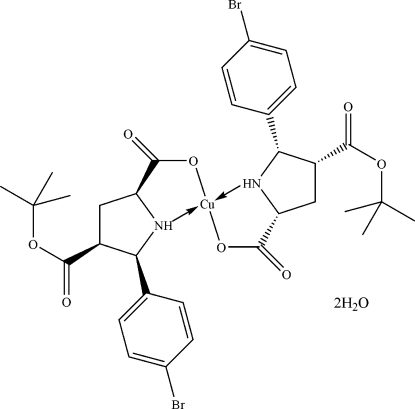


## Experimental

### 

#### Crystal data

[Cu(C_16_H_19_BrNO_4_)_2_]·2H_2_O
*M* *_r_* = 838.04
Monoclinic, 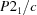

*a* = 15.251 (6) Å
*b* = 10.555 (4) Å
*c* = 10.541 (4) Å
β = 90.423 (6)°
*V* = 1696.9 (11) Å^3^
*Z* = 2
Mo *K*α radiation
μ = 3.06 mm^−1^
*T* = 150 K
0.32 × 0.20 × 0.05 mm

#### Data collection

Bruker SMART APEXII diffractometer
Absorption correction: multi-scan (*SADABS*; Bruker, 2008 ▶) *T*_min_ = 0.441, *T*_max_ = 0.862
12662 measured reflections
3268 independent reflections
2968 reflections with *I* > 2σ(*I*)
*R*_int_ = 0.053

#### Refinement

*R*[*F* ^2^ > 2σ(*F* ^2^)] = 0.066
*wR*(*F* ^2^) = 0.161
*S* = 1.09
3268 reflections
218 parameters
H-atom parameters constrained
Δρ_max_ = 0.75 e Å^−3^
Δρ_min_ = −1.12 e Å^−3^

### 

Data collection: *APEX2* (Bruker, 2008 ▶); cell refinement: *SAINT* (Bruker, 2008 ▶); data reduction: *SAINT*; program(s) used to solve structure: *SHELXTL* (Sheldrick, 2008 ▶); program(s) used to refine structure: *SHELXTL*; molecular graphics: *SHELXTL*; software used to prepare material for publication: *SHELXTL*.

## Supplementary Material

Crystal structure: contains datablock(s) I, global. DOI: 10.1107/S1600536811043893/cv5181sup1.cif

Structure factors: contains datablock(s) I. DOI: 10.1107/S1600536811043893/cv5181Isup2.hkl

Additional supplementary materials:  crystallographic information; 3D view; checkCIF report

## Figures and Tables

**Table 1:** Hydrogen-bond geometry (Å, °)

*D*—H⋯*A*	*D*—H	H⋯*A*	*D*⋯*A*	*D*—H⋯*A*
O5—H51⋯O2	0.82	2.15	2.968 (13)	180
O5—H52⋯O2^i^	0.82	2.18	3.001 (14)	180
N1—H⋯O2^ii^	0.93	1.99	2.916 (9)	173

Symmetry codes: (i) 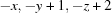; (ii) 
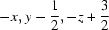
.

**Fig. 1.**
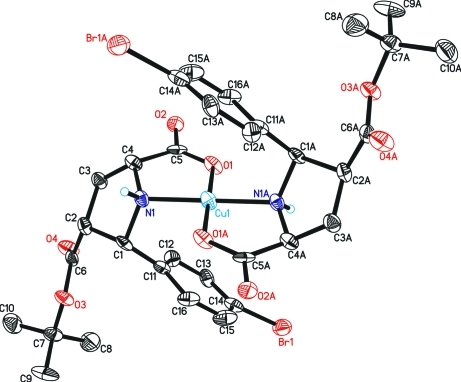
The molecular structure of the title compound, showing the numbering scheme adopted [symmetry code: (A) -x, -y, 2 - z]. Displacement ellipsoids are shown at the 50% probability level. Hydrogen atoms (except amino) are omitted for clarity.

**Fig. 2.**
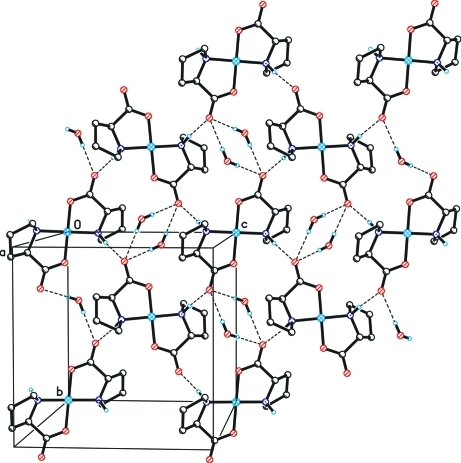
Layers parallel to bc plane. Lateral bromophenyl (–C6H4Br) groups, butoxycarbonyl (–CO2tBu) substituents and hydrogen atoms (except amino and water) are omitted for clarity. Hydrogen bonds are shown as dashed lines.

**Fig. 3.**
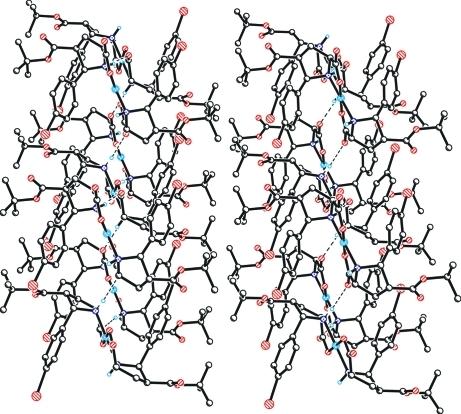
A portion of the crystal packing viewed along axis a. Two parallel laeyers are shown. Hydrogen atoms (except amino and water) are omitted for clarity. Hydrogen bonds are drawn as dashed lines.

## supplementary crystallographic information

### Comment

To the best of our knowledge this is the first X-ray structural analysis of metal complex with 5-arylpyrrolidine-2,4-dicarboxylic acid derivative. In the title compound, an α-amino acid ligand contains additional structural elements - aryl substituent and second carboxylic function, that allows subsequent tuning of complex physico-chemical properties including supramolecular assemblies formation. In the centrosymmetric title complex central copper atom has square-planar coordination environment. There are no additional axial ligands, since both axial positions are shielded by lateral phenyl substituents (Fig. 1). The crystal lattice contains one crystallographycally independent solvent water molecule. Layers parallel to *bc*-plane are formed by N—H···O=C and H_2_O···O=C hydrogen bonds (Table 1, Fig. 2). These layers are linked by weak van-der-Waals interactions between neighbouring *t*-Bu groups (Fig. 3).

### Experimental

(2*S**,4*S**,5*R**)-5-(4-Bromophenyl)- 4-(*tert*-butoxycarbonyl)pyrrolidine-2-carboxylic acid (0.200 g, 0.54 mmol) was suspended in 6 ml of methanol. Anhydrous copper(II) chloride (0.036 g, 0.27 mmol) was added to the suspension in one portion under stirring. The deep blue solution formed immediately. After 1 h the solution was diluted with methanol to 9 m*M* concentration and subjected to slow evaporation at ambient temperature yielding deep blue crystals of the title complex compound.

### Refinement

All hydrogen atoms were placed in calculated positions and refined using a riding model with C—H = 1.00 Å and *U*_iso_(H) = 1.2*U*_eq_(C) for methyne groups; C—H = 0.99 Å and *U*_iso_(H) = 1.2*U*_eq_(C) for methylene groups; C—H = 0.98 Å and *U*_iso_(H) = 1.5*U*_eq_(C) for methyl groups; C—H = 0.95 Å and *U*_iso_(H) = 1.2*U*_eq_(C) for aromatic H atoms; N—H = 0.93 Å and *U*_iso_(H) = 1.2*U*_eq_(N) for NH group; O—H = 0.82 Å and *U*_iso_(H) = 1.2*U*_eq_(O) for water molecule.

The studied crystal was pseudomerohedrally twinned (1 0 0 0 - 1 0 0 0 - 1). The refinement of twin fractions yielded in 0.719 (3)/0.281 (3).

### Crystal data

**Table:**

[Cu(C_16_H_19_BrNO_4_)_2_]·2H_2_O	*F*(000) = 854
*M**_r_* = 838.04	*D*_x_ = 1.640 Mg m^−^^3^
Monoclinic, *P*2_1_/*c*	Mo *K*α radiation, λ = 0.71073 Å
Hall symbol: -P 2ybc	Cell parameters from 4476 reflections
*a* = 15.251 (6) Å	θ = 2.4–25.6°
*b* = 10.555 (4) Å	µ = 3.06 mm^−^^1^
*c* = 10.541 (4) Å	*T* = 150 K
β = 90.423 (6)°	Prism, light-blue
*V* = 1696.9 (11) Å^3^	0.32 × 0.20 × 0.05 mm
*Z* = 2	

### Data collection

**Table:**

Bruker SMART APEXII diffractometer	3268 independent reflections
Radiation source: fine-focus sealed tube	2968 reflections with *I* > 2σ(*I*)
graphite	*R*_int_ = 0.053
Ω scans	θ_max_ = 26.0°, θ_min_ = 1.9°
Absorption correction: multi-scan (*SADABS*; Bruker, 2008)	*h* = −18→18
*T*_min_ = 0.441, *T*_max_ = 0.862	*k* = −13→13
12662 measured reflections	*l* = −13→13

### Refinement

**Table:**

Refinement on *F*^2^	Primary atom site location: structure-invariant direct methods
Least-squares matrix: full	Secondary atom site location: difference Fourier map
*R*[*F*^2^ > 2σ(*F*^2^)] = 0.066	Hydrogen site location: inferred from neighbouring sites
*wR*(*F*^2^) = 0.161	H-atom parameters constrained
*S* = 1.09	*w* = 1/[σ^2^(*F*_o_^2^) + (0.0259*P*)^2^ + 19.8322*P*] where *P* = (*F*_o_^2^ + 2*F*_c_^2^)/3
3268 reflections	(Δ/σ)_max_ < 0.001
218 parameters	Δρ_max_ = 0.75 e Å^−^^3^
0 restraints	Δρ_min_ = −1.12 e Å^−^^3^

### Special details

**Table:**

Geometry. All e.s.d.'s (except the e.s.d. in the dihedral angle between two l.s. planes) are estimated using the full covariance matrix. The cell e.s.d.'s are taken into account individually in the estimation of e.s.d.'s in distances, angles and torsion angles; correlations between e.s.d.'s in cell parameters are only used when they are defined by crystal symmetry. An approximate (isotropic) treatment of cell e.s.d.'s is used for estimating e.s.d.'s involving l.s. planes.
Refinement. Refinement of *F*^2^ against ALL reflections. The weighted *R*-factor *wR* and goodness of fit *S* are based on *F*^2^, conventional *R*-factors *R* are based on *F*, with *F* set to zero for negative *F*^2^. The threshold expression of *F*^2^ > σ(*F*^2^) is used only for calculating *R*-factors(gt) *etc*. and is not relevant to the choice of reflections for refinement. *R*-factors based on *F*^2^ are statistically about twice as large as those based on *F*, and *R*- factors based on ALL data will be even larger.

### Fractional atomic coordinates and isotropic or equivalent isotropic displacement parameters (Å^2^)

**Table:**

	*x*	*y*	*z*	*U*_iso_*/*U*_eq_	
Cu1	0.0000	0.0000	1.0000	0.0234 (3)	
Br1	0.28687 (6)	0.06433 (8)	1.37487 (10)	0.0343 (2)	
N1	0.0529 (4)	−0.0100 (6)	0.8311 (7)	0.0205 (14)	
H	0.0192	−0.0631	0.7799	0.025*	
O1	0.0194 (3)	0.1801 (4)	0.9867 (6)	0.0231 (13)	
O2	0.0442 (4)	0.3320 (6)	0.8483 (6)	0.0328 (15)	
O3	0.3162 (4)	−0.1083 (5)	0.7398 (6)	0.0257 (13)	
O4	0.3285 (4)	0.1004 (5)	0.6961 (7)	0.0321 (15)	
C1	0.1464 (5)	−0.0587 (7)	0.8354 (8)	0.0200 (17)	
H1	0.1455	−0.1526	0.8228	0.024*	
C2	0.1854 (5)	0.0039 (8)	0.7160 (8)	0.0234 (18)	
H2	0.1662	−0.0473	0.6409	0.028*	
C3	0.1413 (6)	0.1349 (8)	0.7051 (8)	0.0254 (18)	
H3A	0.1779	0.2011	0.7458	0.030*	
H3B	0.1312	0.1580	0.6152	0.030*	
C4	0.0542 (5)	0.1190 (8)	0.7750 (8)	0.0235 (17)	
H4	0.0053	0.1253	0.7115	0.028*	
C5	0.0399 (5)	0.2194 (7)	0.8769 (9)	0.0234 (17)	
C6	0.2852 (6)	0.0073 (7)	0.7164 (7)	0.0223 (17)	
C7	0.4129 (5)	−0.1301 (8)	0.7503 (9)	0.0271 (18)	
C8	0.4490 (7)	−0.0602 (10)	0.8626 (11)	0.044 (3)	
H8A	0.4167	−0.0847	0.9388	0.066*	
H8B	0.4429	0.0312	0.8488	0.066*	
H8C	0.5112	−0.0813	0.8738	0.066*	
C9	0.4171 (6)	−0.2711 (9)	0.7707 (12)	0.046 (3)	
H9A	0.3857	−0.2931	0.8484	0.069*	
H9B	0.4785	−0.2975	0.7786	0.069*	
H9C	0.3898	−0.3144	0.6983	0.069*	
C10	0.4562 (6)	−0.0945 (10)	0.6271 (11)	0.041 (2)	
H10A	0.4563	−0.0021	0.6181	0.061*	
H10B	0.4237	−0.1323	0.5561	0.061*	
H10C	0.5167	−0.1258	0.6271	0.061*	
C11	0.1897 (5)	−0.0298 (7)	0.9624 (8)	0.0210 (17)	
C12	0.2329 (5)	0.0837 (7)	0.9889 (9)	0.0243 (18)	
H12	0.2405	0.1448	0.9237	0.029*	
C13	0.2650 (5)	0.1081 (7)	1.1107 (9)	0.0257 (18)	
H13	0.2972	0.1835	1.1268	0.031*	
C14	0.2507 (6)	0.0250 (8)	1.2065 (9)	0.030 (2)	
C15	0.2117 (6)	−0.0916 (8)	1.1805 (10)	0.034 (2)	
H15	0.2060	−0.1530	1.2458	0.041*	
C16	0.1813 (5)	−0.1178 (8)	1.0598 (9)	0.0252 (18)	
H16	0.1543	−0.1972	1.0429	0.030*	
O5	0.0873 (9)	0.5829 (11)	0.9593 (12)	0.107 (4)	
H51	0.0755	0.5136	0.9285	0.128*	
H52	0.0516	0.6063	1.0121	0.128*	

### Atomic displacement parameters (Å^2^)

**Table:**

	*U*^11^	*U*^22^	*U*^33^	*U*^12^	*U*^13^	*U*^23^
Cu1	0.0179 (6)	0.0235 (7)	0.0287 (8)	−0.0049 (6)	0.0057 (7)	−0.0036 (6)
Br1	0.0351 (4)	0.0340 (4)	0.0335 (5)	0.0043 (4)	−0.0055 (5)	0.0007 (4)
N1	0.017 (3)	0.023 (3)	0.022 (3)	−0.001 (3)	0.001 (3)	−0.007 (3)
O1	0.020 (3)	0.008 (2)	0.041 (4)	−0.007 (2)	0.009 (3)	−0.002 (2)
O2	0.046 (4)	0.025 (3)	0.027 (4)	0.013 (3)	0.001 (3)	0.001 (3)
O3	0.026 (3)	0.011 (2)	0.040 (4)	0.002 (2)	0.009 (3)	0.004 (3)
O4	0.027 (3)	0.017 (3)	0.052 (4)	0.000 (3)	0.008 (3)	0.007 (3)
C1	0.020 (4)	0.006 (3)	0.033 (5)	0.001 (3)	0.010 (3)	0.001 (3)
C2	0.023 (4)	0.023 (4)	0.024 (4)	−0.001 (3)	0.008 (3)	−0.003 (3)
C3	0.030 (4)	0.019 (4)	0.028 (5)	0.002 (3)	0.006 (4)	−0.001 (3)
C4	0.017 (4)	0.022 (4)	0.032 (5)	0.001 (3)	0.002 (3)	−0.004 (3)
C5	0.015 (3)	0.020 (4)	0.036 (5)	0.005 (3)	−0.005 (4)	0.011 (4)
C6	0.033 (4)	0.014 (4)	0.020 (4)	0.003 (4)	0.005 (4)	−0.003 (3)
C7	0.024 (4)	0.019 (4)	0.039 (5)	0.001 (3)	0.004 (4)	0.006 (4)
C8	0.041 (5)	0.050 (6)	0.041 (6)	0.006 (5)	−0.011 (5)	−0.008 (5)
C9	0.032 (5)	0.028 (5)	0.077 (8)	0.015 (4)	−0.011 (5)	0.000 (5)
C10	0.029 (5)	0.049 (6)	0.045 (6)	0.004 (4)	0.008 (5)	−0.002 (5)
C11	0.017 (4)	0.015 (4)	0.031 (5)	0.003 (3)	0.005 (3)	0.002 (3)
C12	0.034 (5)	0.011 (3)	0.028 (4)	−0.003 (3)	0.006 (4)	0.001 (3)
C13	0.029 (4)	0.020 (4)	0.028 (5)	−0.003 (3)	0.007 (4)	−0.008 (4)
C14	0.022 (4)	0.026 (4)	0.042 (6)	0.010 (4)	−0.002 (4)	−0.005 (4)
C15	0.027 (4)	0.025 (4)	0.050 (6)	−0.002 (4)	−0.007 (5)	0.012 (4)
C16	0.019 (4)	0.014 (4)	0.042 (5)	−0.006 (3)	−0.004 (4)	0.010 (4)
O5	0.119 (10)	0.092 (8)	0.110 (10)	0.037 (8)	−0.011 (8)	−0.029 (7)

### Geometric parameters (Å, °)

**Table:**

Cu1—O1	1.929 (5)	C7—C8	1.497 (14)
Cu1—O1^i^	1.929 (5)	C7—C9	1.504 (12)
Cu1—N1	1.963 (7)	C7—C10	1.509 (14)
Cu1—N1^i^	1.963 (7)	C8—H8A	0.9800
Br1—C14	1.901 (10)	C8—H8B	0.9800
N1—C4	1.485 (11)	C8—H8C	0.9800
N1—C1	1.516 (10)	C9—H9A	0.9800
N1—H	0.9300	C9—H9B	0.9800
O1—C5	1.271 (11)	C9—H9C	0.9800
O2—C5	1.228 (10)	C10—H10A	0.9800
O3—C6	1.331 (9)	C10—H10B	0.9800
O3—C7	1.495 (10)	C10—H10C	0.9800
O4—C6	1.203 (10)	C11—C16	1.392 (12)
C1—C11	1.519 (12)	C11—C12	1.394 (11)
C1—C2	1.544 (11)	C12—C13	1.395 (13)
C1—H1	1.0000	C12—H12	0.9500
C2—C6	1.523 (12)	C13—C14	1.356 (13)
C2—C3	1.541 (11)	C13—H13	0.9500
C2—H2	1.0000	C14—C15	1.394 (12)
C3—C4	1.534 (11)	C15—C16	1.378 (13)
C3—H3A	0.9900	C15—H15	0.9500
C3—H3B	0.9900	C16—H16	0.9500
C4—C5	1.525 (12)	O5—H51	0.8198
C4—H4	1.0000	O5—H52	0.8200
			
O1—Cu1—O1^i^	180.0	O3—C7—C8	109.9 (7)
O1—Cu1—N1	85.6 (3)	O3—C7—C9	101.8 (7)
O1^i^—Cu1—N1	94.4 (3)	C8—C7—C9	111.1 (9)
O1—Cu1—N1^i^	94.4 (3)	O3—C7—C10	109.6 (7)
O1^i^—Cu1—N1^i^	85.6 (3)	C8—C7—C10	113.4 (8)
N1—Cu1—N1^i^	179.999 (1)	C9—C7—C10	110.6 (8)
C4—N1—C1	107.9 (6)	C7—C8—H8A	109.5
C4—N1—Cu1	108.6 (5)	C7—C8—H8B	109.5
C1—N1—Cu1	112.6 (5)	H8A—C8—H8B	109.5
C4—N1—H	109.3	C7—C8—H8C	109.5
C1—N1—H	109.3	H8A—C8—H8C	109.5
Cu1—N1—H	109.3	H8B—C8—H8C	109.5
C5—O1—Cu1	115.2 (5)	C7—C9—H9A	109.5
C6—O3—C7	120.2 (6)	C7—C9—H9B	109.5
N1—C1—C11	111.2 (6)	H9A—C9—H9B	109.5
N1—C1—C2	101.5 (6)	C7—C9—H9C	109.5
C11—C1—C2	117.7 (6)	H9A—C9—H9C	109.5
N1—C1—H1	108.7	H9B—C9—H9C	109.5
C11—C1—H1	108.7	C7—C10—H10A	109.5
C2—C1—H1	108.7	C7—C10—H10B	109.5
C6—C2—C3	114.5 (7)	H10A—C10—H10B	109.5
C6—C2—C1	113.5 (7)	C7—C10—H10C	109.5
C3—C2—C1	105.9 (6)	H10A—C10—H10C	109.5
C6—C2—H2	107.5	H10B—C10—H10C	109.5
C3—C2—H2	107.5	C16—C11—C12	118.1 (8)
C1—C2—H2	107.5	C16—C11—C1	118.4 (7)
C4—C3—C2	104.2 (6)	C12—C11—C1	123.4 (7)
C4—C3—H3A	110.9	C11—C12—C13	120.3 (8)
C2—C3—H3A	110.9	C11—C12—H12	119.8
C4—C3—H3B	110.9	C13—C12—H12	119.8
C2—C3—H3B	110.9	C14—C13—C12	120.6 (8)
H3A—C3—H3B	108.9	C14—C13—H13	119.7
N1—C4—C5	110.8 (7)	C12—C13—H13	119.7
N1—C4—C3	107.8 (6)	C13—C14—C15	119.7 (9)
C5—C4—C3	113.0 (7)	C13—C14—Br1	120.4 (7)
N1—C4—H4	108.4	C15—C14—Br1	119.8 (7)
C5—C4—H4	108.4	C16—C15—C14	119.9 (8)
C3—C4—H4	108.4	C16—C15—H15	120.0
O2—C5—O1	123.6 (8)	C14—C15—H15	120.0
O2—C5—C4	119.4 (8)	C15—C16—C11	121.0 (8)
O1—C5—C4	116.9 (7)	C15—C16—H16	119.5
O4—C6—O3	125.9 (8)	C11—C16—H16	119.5
O4—C6—C2	124.6 (8)	H51—O5—H52	113.1
O3—C6—C2	109.4 (7)		

Symmetry codes: (i) −*x*, −*y*, −*z*+2.

### Hydrogen-bond geometry (Å, °)

**Table:**

*D*—H···*A*	*D*—H	H···*A*	*D*···*A*	*D*—H···*A*
O5—H51···O2	0.82	2.15	2.968 (13)	180.
O5—H52···O2^ii^	0.82	2.18	3.001 (14)	180.
N1—H···O2^iii^	0.93	1.99	2.916 (9)	173.

Symmetry codes: (ii) −*x*, −*y*+1, −*z*+2; (iii) −*x*, *y*−1/2, −*z*+3/2.
